# Short-Term Efficacy and Adverse Effects of Sulfasalazine in the Management of Axial Spondyloarthropathy

**DOI:** 10.7759/cureus.49978

**Published:** 2023-12-05

**Authors:** Gauravi Deshpande, Archana Sonawale, Alhad Mulkalwar, Hritvik Jain, Aman Goyal

**Affiliations:** 1 Internal Medicine, Seth Gordhandas Sunderdas Medical College and King Edward Memorial Hospital, Mumbai, IND; 2 Internal Medicine, All India Institute of Medical Sciences (AIIMS) Jodhpur, Jodhpur, IND

**Keywords:** rheumatic diseases, clinical rheumatology, targeted biologics, arthritis, (nsaid) non-steroidal anti-inflammatory drugs, sulfasalazine, ankylosing spondylarthritis

## Abstract

Introduction: Ankylosing spondylitis (AS) is an inflammatory spondyloarthropathy that involves the sacroiliac joints and the axial skeleton. Sulfasalazine's efficacy in treating the axial symptoms of AS has been a subject of controversy.

Methods: This prospective observational study recruited AS patients and categorized them into two groups: the first group had AS for less than or equal to four years and the second group had AS for more than four years. Erythrocytic sedimentation rate (ESR) and C-reactive protein (CRP) levels were recorded at baseline and at six-month follow-up. Disease severity was assessed using the ankylosing spondylitis disease activity score (ASDAS), Bath ankylosing spondylitis disease activity index (BASDAI) score, and Bath ankylosing spondylitis functional index (BASFI) score.

Results: A total of 33 patients diagnosed with AS were recruited in this study, mostly males (88%) and within 21-30 years of age. ESR and CRP values were measured at baseline and at six months post-treatment with sulfasalazine. Mean ESR and mean CRP values showed a statistically significant reduction of 43.5% (p=0.001) and 58.45% (p=0.0012) respectively, at the 6-month follow-up. Four patients (12.12%) reported gastrointestinal intolerance. The mean reduction in the ASDAS score was 24% (p=0.002), the BASDAI score was 40.08% (p=0.001), and the BASFI score was 39.54% (p=0.01). Additionally, the duration of symptoms did not appear to influence with efficacy of sulfasalazine.

Discussion: Sulfasalazine is a safe alternative therapy for patients with AS who cannot afford biologics, due to its reasonable short-term efficacy, good tolerability, cost-effective nature, and low incidence of adverse effects.

## Introduction

Ankylosing spondylitis (AS), a spondyloarthropathy, is a chronic inflammatory disorder involving multiple systems namely cardiovascular, respiratory, musculoskeletal, ophthalmic, and skin. AS primarily affects the sacroiliac joints and the axial skeleton. The etiology of AS is not entirely understood, however, a strong genetic predisposition exists in the causation of AS. A direct causal association has been linked between AS and the HLA-B27 gene [1]. The primary pathological process in AS is enthesitis with chronic inflammation, which involves the release of inflammatory cytokines like IL-17 and IL-23.

Sulfasalazine belongs to the class of disease-modifying anti-rheumatic drugs (DMARDs). Sulfasalazine is a pro-drug that forms its active metabolites, sulfapyridine and 5-aminosalicylic acid (5-ASA) when acted upon by colonic bacteria [1]. 5-ASA has anti-inflammatory properties as it is known to inhibit leukotriene synthesis and lipoxygenase. It is the main constituent responsible for the pharmacological effects of sulfasalazine. AS is an inflammatory arthropathy that can present with both axial and peripheral symptoms. Sulfasalazine has demonstrated efficacy in treating peripheral arthritis associated with AS, yet its impact on the axial symptoms of AS remains unexplored in the Indian population. This gap persists due to several factors such as considerably higher cost of the medication, underdiagnosed patient population, and other barriers [2,3]. However, in clinical practice, sulfasalazine has also shown reasonable efficacy in improving the backache symptoms associated with axial spondyloarthropathy, especially in patients in earlier phases of the disease with a disease duration of less than four years, or younger patients [4].

While non-steroidal anti-inflammatory drugs (NSAIDs) are conventionally considered to be the standard of care in patients with AS, biologics like anti-tumor necrosis factor-ɑ (TNF- ɑ) are now the first-line therapy for patients with axial spondyloarthropathy as per the Assessment of Spondyloarthritis International Society/European League Against Rheumatism (ASAS/EULAR) [5,6]. One of the major drawbacks of using anti-TNF-ɑ drugs in developing countries is the cost associated with the usage of biologics. This prospective observational study aimed at investigating the efficacy, adverse events, and clinical outcomes achieved using sulfasalazine in axial spondyloarthropathy.

## Materials and methods

Study design

This study was a prospective observational study conducted over 24 months on a sample size of 33 patients. This study was conducted at a government tertiary care referral center in Mumbai, India.

Inclusion criteria

Patients between the ages of 18 and 45 years, who fulfilled the ASAS for axial spondyloarthritis (SpA) and were about to commence treatment with sulfasalazine, were included in this study [7]. ASAS for axial SpA classification in patients with back pain ≥ three months and age of onset < 45 years, includes either sacroiliitis features on imaging with ≥ one SpA feature or HLA-B27 positivity with ≥ two other SpA features.

Exclusion criteria

Pregnant and lactating women, patients allergic to sulfa drugs, patients already taking sulfasalazine, and those who required or could afford biologic drugs were excluded from the study. No cases were lost to follow-up.

Study procedure

Patients who fulfilled the inclusion criteria were approached for voluntary participation in the study after taking an informed written consent. A semi-structured questionnaire was used to record the demographic details of the patients, past medical history, and clinical examination findings. The patient’s current complaints and the total duration of their symptoms were assessed. Patients were divided into two groups based on their duration of symptoms, the first group had symptoms lasting less than or equal to four years, and the second group had symptoms lasting for more than four years. Erythrocytic sedimentation rate (ESR) and C-reactive protein (CRP) levels were recorded at baseline and at six months. Baseline ankylosing spondylitis disease activity score (ASDAS), Bath ankylosing spondylitis disease activity index (BASDAI) score, and Bath ankylosing spondylitis functional index (BASFI) score were calculated at the beginning and again at six months [8-10]. Patients were also followed up at three months. Any adverse events related to sulfasalazine reported by the patients were also reported.

An ASDAS score of less than 1.3 was defined as an inactive disease, a score between 1.3 and 2.1 was classified as moderate disease activity, a score between 2.1 and 3.5 indicated high disease activity and a score higher than 3.5 represented a very high disease activity. A reduction in ASDAS score of less than 1.1 was considered as no improvement, a reduction of greater than or equal to 1.1 to 2.0 was considered a clinically significant improvement, and a reduction of more than 2.0 in the score was considered a major improvement. Patients with BASDAI scores less than or equal to 4 were classified as having mild disease activity, while those with a score higher than 4 were said to have high disease activity. BASFI score is related to activities of daily living and is ranging from 0 (no functional impairments) to 10 (maximal impairment). 

Statistical analysis

The data of the statistical parameters were entered into an Excel sheet and analyzed using the Wilcoxon signed-rank test and paired t-test. All p-values less than 0.05 were considered statistically significant with a confidence interval of 95%. All statistical analyses were performed with the use of the Statistical Package for Social Sciences (SPSS) software version 28 (SPSS, IBM).

Ethical considerations

Ethical approval was granted by the Institutional Ethics Committee (IEC) of Seth Gordhandas Medical College and King Edward Memorial Hospital, Mumbai, India (IEC No.: IEC(I)/OUT/349/2018), Furthermore, this study followed the principles of the Declaration of Helsinki. All participants were recruited only after taking their written informed consent.

## Results

Demographic details

A total of 33 patients were recruited for this study. Most of the patients (n = 17; 51.5%) were in the age group of 21-30, and the majority of patients were male (n = 29; 88%) (Table 1). 

**Table 1:** Descriptive statistics of demographic details. Statistics are represented as numbers (percentages).

Variable	Frequency; n(%)
Age Group (in years)	
18 - 20	6 (18.2 %)
21 - 30	17 (51.5 %)
31 - 40	9 (27.3 %)
41 - 50	1 (3.0 %)
Gender	
Male	29 (88.0 %)
Female	4 (12.0 %)

Clinical profile

At presentation, out of the 33 recruited patients, 21 patients had a duration of symptoms for less than or equal to four years, whereas 12 patients had a duration of symptoms for more than four years. In this study, all patients (n = 33; 100%) presented with inflammatory back pain as a clinical feature, the next most common clinical symptom was enthesitis (n = 16; 48.5%). The most common adverse effect seen in patients consuming sulfasalazine was gastrointestinal intolerance in the form of nausea, vomiting, or abdominal pain, seen in 12.12% of the patients (Table 2). The mean ESR value at the time of admission for the patients was 36.152 mm/hr, and at the six-month follow-up, it was 20.42 mm/hr (p = 0.001). Thus, there was a decrease of about 43.5% in the mean ESR at six months compared to baseline. The mean baseline CRP value was 19.47 mg/dl which decreased to 8.09 mg/dl at the six-month follow-up, which is a statistically significant decrease by 58.45% from its initial mean value (p = 0.0012).

**Table 2:** Signs, symptoms and adverse effects to sulfasalazine. Statistics are represented as numbers (percentages).

Variable	Frequency; n(%)
Presence of signs and symptoms	
Inflammatory back pain	33 (100 %)
Enthesitis	16 (48.9 %)
Arthritis	7 (21.2 %)
Uveitis	4 (12.1%)
Dactylitis	0
Psoriasis	0
Crohn’s disease	0
Adverse effects to sulfasalazine	
Gastrointestinal intolerance	4 (12.1%)
Dermatologic rash	1 (3.0 %)
Hematological adverse events including anemia, thrombocytopenia, and neutropenia	0
Drug-induced hepatitis	0

Efficacy of sulfasalazine based on ASDAS score

Upon initial presentation, all patients exhibited a very high disease activity as per the ASDAS score. After six months of treatment with sulfasalazine, approximately 36% experienced an improvement in their ASDAS scores, transitioning from a “very high disease activity” index to a “high disease activity” index. The mean ASDAS score at baseline was 5.304, and it decreased to 3.896 after six months of sulfasalazine treatment. The mean score reduction was 24%, which was statistically significant with p = 0.002. Table 3 illustrates the improvements observed in patients using sulfasalazine at the six-month mark, according to the ASDAS score. The decrease in the ASDAS score for patients with a disease duration of less than four years was 30.67%, whereas the other group which comprised of patients with symptoms more than four years exhibited a decrease of 19.56%. However, the magnitude of the two differences was not statistically significant (p = 0.407).

**Table 3:** Improvement in ASDAS score after treatment with sulfasalazine for six months.

Variable	Frequency; n (%)
Major improvement	3 (9.1 %)
Clinically significant improvement	20 (60.6 %)
No improvement	10 (30.3 %)

The need for consumption of concomitant NSAIDs along with sulfasalazine was less in most patients who showed clinically significant improvement. Among the patients with major clinical improvement, 55% required NSAIDs only once a month. On the other hand, a majority of the patients with no improvement required high dosages of NSAIDs. Specifically, 30% of such patients required NSAIDs once a day, while 20% required NSAIDs twice a day (Table 4).

**Table 4:** Correlation between the frequency of consumption of NSAIDs and improvements in the ASDAS score. NSAID: Non-steroidal anti-inflammatory drug; ASDAS: Ankylosing spondylitis disease activity score

Frequency of NSAIDs consumption	Total number of patients (n)	Clinically significant improvement (n)	Major improvement (n)	No improvement (n)
Once a month	15	11	1	3
Twice a month	1	1	0	0
Once a week	3	2	1	0
2 times a week	1	0	1	0
3 times a week	1	1	0	0
4 times a week	1	1	0	0
5 times a week	3	1	0	2
Once a day	4	1	0	3
Twice a day	4	2	0	2

Efficacy of sulfasalazine based on the BASDAI score

At the initial presentation, all patients had high disease activity. After six months, 67% of patients had a score of 4 or less, while 33% still experienced high disease activity. The mean BASDAI score at the initial presentation was 5.761, and at the six-month follow-up, it was 3.452. After six months of using sulfasalazine, there was a significant reduction in the score by 40.08% (p = 0.001). The decrease in the BASDAI score for patients with a disease duration of less than 4 years was 46.8%, whereas the other group, comprising patients with a symptom duration of more than four years, exhibited a decrease of 29.22%. However, the magnitude of the two differences was not statistically significant (p = 0.238).

Efficacy of sulfasalazine based on the BASFI score

The mean BASFI scores at baseline and at six months were 5.118 and 3.094, respectively. The mean reduction in the BASFI score was 39.54%, which was statistically significant (p = 0.01). The decrease in the BASFI score for patients with a disease duration of less than four years was 48.6%, whereas the other group, comprising patients with a symptom duration of more than four years, exhibited a decrease of 26.24%. However, the magnitude of the two differences was not statistically significant (p = 0.134).

## Discussion

In this study, 51.51% of the patients were in the age group of 20-30 years. In a study conducted by Sharma et al., the mean age of patients in the treatment group was 31.32 ± 10.12 years [11]. Furthermore, in our study, 63.63% of the patients presented with a duration of less than four years. In the study conducted by Sharma et al., 41.93% of the patients in the treatment group (n=31) had a disease duration of four to seven years [11]. 

In our study, all the patients presented with inflammatory back pain, with enthesitis being the next most common symptom. In a study conducted by Braun et al., 91% of patients experienced pain in the lumbar spine, 39% had thoracic spine involvement, and 33% reported cervical spine pain [12]. Enthesitis was observed in 50% of patients, followed by dactylitis and uveitis, seen in 14% and 3% of patients, respectively. 

Gastrointestinal disturbance occurred in 12.12% of our patients as a side effect of sulfasalazine, manifesting as nausea, vomiting, and abdominal pain. In studies conducted by Braun et al. and Nissila et al., the rates of gastrointestinal disturbance due to the use of sulfasalazine were 17% and 14%, respectively [12,13]. The study by Braun et al. demonstrated no significant reduction in mean ESR and CRP levels with the use of sulfasalazine [12]. However, studies conducted by Nissila et al., Clegg et al., and Sharma et al. did show this reduction, consistent with our own findings [11,13,14].

Some rare adverse effects of sulfasalazine have been reported in patients with spondyloarthropathies and other inflammatory arthritis. Caterson et al. reported sulfasalazine-induced myopathy in a case of acute spondyloarthritis [15]. Karmakar et al reported lung toxicity when treated with sulfasalazine [16]. Drug reaction with eosinophilia and systemic symptoms (DRESS) has also been reported to be induced by sulfasalazine in a case of rheumatoid arthritis by Bejia et al., similar findings have been reported by Yesilova et al. and Sah et al. in a case of seronegative spondyloarthritis. Sulfasalazine-induced aplastic anemia has also been reported in a patient with rheumatoid arthritis by Nurmohamed et al. 2000 [17-20].

In this current study, we observed a clinically significant improvement based on the reduction in ASDAS scores in 61% of patients, which is similar to the 66.7% observed in the study conducted by Sharma et al. [11]. Additionally, our study showed a 40.08% decrease in the BASDAI score after six months of treatment compared to the baseline. In Sharma et al.'s study, they reported a 61% decrease in the BASDAI score. However, Braun et al. found no significant change in the BASDAI score when treated with sulfasalazine. Moreover, in our study, a 39.54% decrease in the BASFI score was observed at six months compared to baseline during sulfasalazine treatment, consistent with the findings reported by Sharma et al., but no such association was found by Braun et al. [12]. 

The study conducted by Sharma et al. divided patients into three groups based on the duration of the disease: less than four years, four to seven years, and more than seven years. They found the mean reduction in the ASDAS score to be significant in the treatment group compared to the placebo group [11]. In our study, we categorized the patients into two groups: those with a symptom duration of less than or equal to four years and those with more than four years. However, we observed no significant reduction in any of the three scoring systems. 

In our study, the requirement for NSAID usage was lower in most patients who exhibited clinically significant improvement. According to the study conducted by Braun et al., the necessity for NSAIDs had significantly decreased in the treatment group when compared to the placebo group after three months of sulfasalazine treatment [12]. This demonstrates that in patients for whom sulfasalazine is effective, the need for concurrent NSAIDs can be diminished. As a result, this reduction helps to minimize side effects like gastrointestinal disturbances and bleeding complications associated with the consumption of high doses of NSAIDs. 

For rehabilitation in patients with AS, acupuncture combined with dynamic moxibustion has shown statistically significant efficacy (p<0.05) in improving the clinical symptoms of AS, potentially as an adjunctive modality [21]. Electronic focusing high-energy extracorporeal shockwave combined with conventional treatment (NSAIDs and sulfasalazine) has shown appreciable clinical effects in relieving pain and improving clinical outcomes in patients with AS [22]. In a recent clinical trial by Rajaei et al., 40 mg of intra-sacroiliac joint methylprednisolone injection has shown efficacy in pain relief and clinical improvement of joint function in patients with spondyloarthropathies [23]. These therapies can be considered viable as an adjunct to treatment with sulfasalazine for patients with AS, though the current study did not determine their efficacy and outcomes.

Limitations

There were several limitations in this current study. First, the sample size was relatively small, and the sampling was conducted at a single centre, potentially limiting the generalizability of the findings. Second, a longer follow-up duration would hold greater clinical relevance for a chronic disease like axial spondyloarthropathy. Third, most of our patients were male. Fourth, our study did not utilize indexes to measure the effect of sulfasalazine on improving spinal mobility, like the Bath ankylosing spondylitis metrology index [24]. Lastly, large-scale randomized controlled trials comparing the efficacy of sulfasalazine to other first-line agents will be required to solidify our findings.

## Conclusions

Sulfasalazine due to its reasonable efficacy, good tolerability, cost-effective nature, and low incidence of adverse effects, sulfasalazine can be considered as an alternative therapy for patients with axial spondyloarthropathy who cannot afford biologics, particularly in developing nations. Future rheumatology research should aim at large multicentric clinical trials evaluating the efficacy of sulfasalazine in developing nations and find more affordable biologics for AS patients unresponsive to the conventional treatment of NSAIDs, sulfasalazine and methotrexate.

## References

[1] van Tubergen A, Weber U (2012). Diagnosis and classification in spondyloarthritis: identifying a chameleon. Nat Rev Rheumatol.

[2] Deodhar A, Strand V, Kay J, Braun J (2016). The term 'non-radiographic axial spondyloarthritis' is much more important to classify than to diagnose patients with axial spondyloarthritis. Ann Rheum Dis.

[3] Kiltz U, Baraliakos X, Karakostas P (2012). Do patients with non-radiographic axial spondylarthritis differ from patients with ankylosing spondylitis?. Arthritis Care Res (Hoboken).

[4] Brophy S, Calin A (2001). Ankylosing spondylitis: Interaction between genes, joints, age at onset, and disease expression. J Rheumatol.

[5] Brewerton DA (1978). Inherited susceptibility to rheumatic disease. J R Soc Med.

[6] Maxwell LJ, Zochling J, Boonen A (2015). TNF-alpha inhibitors for ankylosing spondylitis. Cochrane Database Syst Rev.

[7] Machado P, Landewé R, Lie E, Kvien TK, Braun J, Baker D, van der Heijde D (2011). Ankylosing spondylitis disease activity score (ASDAS): defining cut-off values for disease activity states and improvement scores. Ann Rheum Dis.

[8] Calin A, Garrett S, Whitelock H, Kennedy LG, O'Hea J, Mallorie P, Jenkinson T (1994). A new approach to defining functional ability in ankylosing spondylitis: the development of the Bath ankylosing spondylitis functional index. J Rheumatol.

[9] Garrett S, Jenkinson T, Kennedy LG, Whitelock H, Gaisford P, Calin A (1994). A new approach to defining disease status in ankylosing spondylitis: the Bath ankylosing spondylitis disease activity index. J Rheumatol.

[10] Rudwaleit M, van der Heijde D, Landewé R (2011). The Assessment of SpondyloArthritis International Society classification criteria for peripheral spondyloarthritis and for spondyloarthritis in general. Ann Rheum Dis.

[11] Khanna Sharma S, Kadiyala V, Naidu G, Dhir V (2018). A randomized controlled trial to study the efficacy of sulfasalazine for axial disease in ankylosing spondylitis. Int J Rheum Dis.

[12] Braun J, Zochling J, Baraliakos X (2006). Efficacy of sulfasalazine in patients with inflammatory back pain due to undifferentiated spondyloarthritis and early ankylosing spondylitis: a multicentre randomised controlled trial. Ann Rheum Dis.

[13] Nissilä M, Lehtinen K, Leirisalo-Repo M, Luukkainen R, Mutru O, Yli-Kerttula U (1988). Sulfasalazine in the treatment of ankylosing spondylitis. A twenty-six-week, placebo-controlled clinical trial. Arthritis Rheum.

[14] Clegg DO, Reda DJ, Abdellatif M (1999). Comparison of sulfasalazine and placebo for the treatment of axial and peripheral articular manifestations of the seronegative spondylarthropathies: a Department of Veterans Affairs cooperative study. Arthritis Rheum.

[15] Caterson HC, Youssef P (2022). Sulfasalazine-induced myopathy in a patient with an acute spondyloarthritis. Intern Med J.

[16] Karmakar GC, Wong CA, Horwood F, Anderson G (2010). Sulphasalazine lung toxicity: report of two cases. N Z Med J.

[17] Bejia I, Ben Hammouda S, Riahi K, Zinelabidine F, Mediouni B, Touzi M, Bergaoui N (2006). DRESS syndrome induced by sulphasalazine in rheumatoid arthritis. Joint Bone Spine.

[18] Yeşilova Z, Kantarcioğlu M, Erçin CN (2009). Sulfasalazine-induced hypersensitivity: a case report of DRESS syndrome. Turk J Gastroenterol.

[19] Sah N, Ramaiah B, Koneri R (2021). Sulfasalazine-induced drug rash with eosinophilia and systemic symptoms syndrome in a seronegative spondyloarthritis patient: a case report. Indian J Pharmacol.

[20] Nurmohamed MT, Soesan M, van Oers MH, Dijkmans BA, van Soesbergen RM (2000). Cyclosporin for sulphasalazine-induced aplastic anaemia in a patient with early rheumatoid arthritis. Rheumatology (Oxford).

[21] Chen ZL, Pang GS (2016). Acupuncture assisted by dynamic moxibustion for adult ankylosing spondylitis at early-to-mid stage (Article in Chinese). Zhongguo Zhen Jiu.

[22] Zhang WY, Chen XT, Wang XQ, Zhang LL, Luo J (2021). Electronic focusing high-nergy extracorporeal shockwave combined with conventional oral medicine for the treatment of ankylosing spondylitis (Article in Chinese). Zhongguo Gu Shang.

[23] Rajaei E, Bavieh K, Mowla K, Ghorbani A, Movaffagh N (2022). The impact of intra-sacroiliac joint methylprednisolone injection in the recovery of patients with spondyloarthropathy: a randomized controlled trial. Reumatologia.

[24] Zochling J (2011). Measures of symptoms and disease status in ankylosing spondylitis: Ankylosing Spondylitis Disease Activity Score (ASDAS), Ankylosing Spondylitis Quality of Life Scale (ASQoL), Bath Ankylosing Spondylitis Disease Activity Index (BASDAI), Bath Ankylosing Spondylitis Functional Index (BASFI), Bath Ankylosing Spondylitis Global Score (BAS-G), Bath Ankylosing Spondylitis Metrology Index (BASMI), Dougados Functional Index (DFI), and Health Assessment Questionnaire for the Spondylarthropathies (HAQ-S). Arthritis Care Res (Hoboken).

